# Perspectives from the 2024 School on Neuropharmacology Research and Drug Development in Harare, Zimbabwe: insights from participants

**DOI:** 10.1242/bio.062217

**Published:** 2026-06-22

**Authors:** John Senanu, Bertrand Yuwong Wanyu, Jessica Burns, Victor Bassey Archibong, Rosa M. Chinheya, Nji S. Ombel Musa, Shingirayi Zengeni, Asmaa Haj-Khlifa, Kehinde Oluwaseyi Adeniji, Rhoda Mama Kolo, Mooki Caroline, Fatma Zahra Sakhri, Elias Chipofya

**Affiliations:** ^1^ Department of Biochemistry, University of Cape Coast, P. O. Box 426, Cape Coast, Ghana; ^2^ Department of Animal Biology and Conservation, University of Buea, P. O. Box 63, Buea, Cameroon; ^3^ Division of Human Genetics and Molecular Biology, Stellenbosch University, Private Bag X1, Matieland, Stellenbosch 7602, South Africa; ^4^ Department of Human Anatomy, University of Rwanda, P. O. Box 4285, Kigali, Rwanda; ^5^ Department of Pharmacology, University of the Free State, P. O. Box 339, Bloemfontein 9300, South Africa; ^6^ Department of Pharmacy and Pharmaceutical Sciences, University of Zimbabwe, P. O. Box MP167, Mount Pleasant, Harare, Zimbabwe; ^7^ Faculty of Sciences Semlalia, Cadi Ayyad University, B. P. 511-4000 Marrakech, Morocco; ^8^ Department of Anatomy, Babcock University, 121003 Ilishan-Remo, Ogun, Nigeria; ^9^ Department of Anatomy, University of Ilorin, 240003 Kwara, Ilorin South, Nigeria; ^10^ DSI/Preclinical Drug Development Platform, North-West University, Private Bag X6001, 2520 Potchefstroom, South Africa; ^11^ Laboratoire d' Obtention de Substances Therapeutiques, National Research Center of Biotechnology, UV 03, BP E73, 25000 Constantine, Algeria; ^12^ National Higher School of Biotechnology, P. O. Box E66, 25100 Constantine, Algeria; ^13^ Department of Biotechnology and Consumer Sciences, Cape Peninsula University of Technology, P. O. Box 1906, Bellville, 7535 Cape Town, South Africa

**Keywords:** Neuropharmacology, Africa, Drug discovery, Medicinal plants, Research training, IBRO

## Abstract

Sub-Saharan Africa is experiencing a rising burden of neurological and mental disorders. The International Brain Research Organization (IBRO)-funded 2nd School on Neuropharmacology Research and Drug Development (NRDD) was held in Harare, Zimbabwe, 2024. The meeting aimed to (1) provide a theoretical foundation in neuropharmacology; (2) develop skills in experimental design, pharmacokinetics, and drug evaluation; (3) foster interdisciplinary collaboration and networking; and (4) promote cultural and scientific exchange. Thirteen participants from eight African countries attended the 1-week program. This Meeting Review evaluates the extent to which the meeting achieved its goals, drawing on structured reflections from all participants, and describes the scientific, networking, cultural, and logistical dimensions of the experience. The school successfully equipped participants with foundational knowledge in neuropharmacology and drug development. Future editions would benefit from extended hands-on sessions and structured post-program follow-up.

## Teaser

**Summary:** Through participant reflections, this Meeting Review evaluates the impact of a pan-African neuropharmacology school, highlighting effective training models, mentorship, and capacity building for drug discovery.

## Introduction

Globally, neurological and mental disorders are a leading cause of disability-adjusted life years (GBD 2019 Mental Disorders Collaborators, 2022; GBD 2021 Nervous System Disorders Collaborators, 2024). Between 2000 and 2015, the population of the African continent grew by 49%, accompanied by a rise in neurological and mental disorders (Sankoh et al., 2018). This increase is attributable to poverty, malnutrition, infectious diseases, and limited access to neuropsychiatric care (Bos et al., 2006). There is, therefore, an urgent call for African scientists to harness the continent's rich medicinal plant resources as a complement to conventional therapies (Kinda et al., 2017; Amoateng et al., 2018).

Africa's biodiversity offers vast potential for drug discovery, yet many researchers lack the specialized pharmacological knowledge needed to characterize neuroactive compounds rigorously (Mahomoodally, 2013; Burns et al., 2023; Ger et al., 2024). This deficiency represents a significant barrier to advancing the drug discovery pipeline.

To address this gap, the International Brain Research Organization (IBRO)-funded 2nd School on Neuropharmacology Research and Drug Development (NRDD) was held in Harare, Zimbabwe, in 2024. Thirteen early-career researchers from eight African countries attended the 1-week residential program, hosted by the University of Zimbabwe and supported by a faculty of seven internationally recognized experts (see Acknowledgements). Participants were selected through a competitive application process, with priority given to those working on natural product-based research or neurological disease in an African context.

The school was designed with four overarching goals: (1) to provide a robust theoretical foundation in pharmacological principles relevant to neuroactive compound research; (2) to develop skills in experimental design, pharmacokinetics, and preclinical evaluation; (3) to foster interdisciplinary collaboration and professional networking; and (4) to enrich the experience through cultural and social exchange in the host country.

This narrative Meeting Review evaluates the extent to which these goals were achieved, drawing on written reflections collected from all 13 participants at the close of the program. It encompasses the scientific and educational impact of the program, networking and collaboration fostered, cultural exchanges that enriched the experience and logistical aspects of the school's organization.

## Meeting overview and rationale

The school was a 1-week intensive residential program held at Manna Resorts on the outskirts of Harare. The 13 participants represented eight African countries – Algeria, Cameroon, Ghana, Morocco, Nigeria, Rwanda, South Africa, and Zimbabwe – and diverse disciplines including biochemistry, anatomy, pharmacology, microbiology, and chemistry. Participants ranged from advanced PhD students to early-career postdoctoral researchers, many of whom had limited prior formal training in pharmacology.

This diversity was central to the school's educational philosophy. Unlike a conventional university pharmacology course – which typically follows a fixed curriculum delivered to a homogeneous student cohort with limited cross-national interaction – this school was specifically designed to address gaps that standard university training does not fill. The school was unique in (1) its focus on African medicinal plants and the specific challenges of conducting neuropharmacology research in resource-limited African contexts; (2) integration of active learning methodologies [problem-based learning (PBL), team-based learning (TBL), facilitated discussions] rather than passive lecture delivery alone; (3) use of participants' own ongoing research projects as the basis for applied learning; (4) deliberate cultivation of a pan-African scientific community through cross-national collaboration and mentorship; and (5) coverage of the complete drug development pipeline – from plant extraction to preclinical evaluation – within a single cohesive program.

The curriculum was structured around three thematic pillars. The first covered foundational concepts: functional neuroanatomy, drug-receptor interactions, pharmacokinetics and pharmacodynamics, and mechanisms of drug metabolism. The second focused on research skills: identification of lead compounds from natural sources, best practices for plant extraction, experimental design using *in vitro* and *in vivo* models, dose-response analysis, and toxicity assessment. The third addressed broader scientific development: research ethics, scientific writing, science communication, and grant funding guidance.

Active-learning methodologies were integrated throughout. PBL sessions required teams to analyze real-world drug discovery challenges, while TBL modules structured group problem solving around pharmacological case studies. Participants also presented their own research for peer and faculty feedback. This pedagogical approach was designed to promote transferable skills rather than rote knowledge acquisition – a deliberate distinction from passive, lecture-only formats common in many graduate programs.

## Scientific and educational impact

One of the primary goals of the school was to provide participants – many of whom had limited prior pharmacology training – with a rigorous grounding in neuropharmacological principles. Written reflections from all 13 participants indicate that this goal was substantially achieved, though with areas identified for improvement.

Across reflections, sessions most frequently cited as impactful were those on pharmacokinetics and pharmacodynamics, experimental models, and lead compound identification – reflecting their direct relevance to participants' own research on natural products and preclinical studies.

*The program's diverse lectures provided comprehensive insights into three key areas. The series on Neuropharmacology: Principles and Practical Applications was particularly relevant … as many students, including myself, work with plant extracts. This series emphasized the importance of understanding effective doses, optimal administration routes, and distinguishing between potency and efficacy. It also highlighted the need to consider whether compounds can cross the blood-brain barrier when researching brain-related effects.*

Fatma Zahra Sakhri, PhD, Algeria

*The sessions on experimental models emphasized the importance of selecting appropriate models for specific research questions, considering the validity types: face, construct, and predictive validity.*

Fatma Zahra Sakhri, PhD, Algeria

The opportunity to present research and receive structured feedback was a distinctive feature of the program:

*A valuable highlight was presenting my research and receiving constructive feedback. Despite my initial apprehension towards pharmacology, the facilitators made the sessions enjoyable and helped me overcome my phobia.*

Victor Bassey Archibong, PhD, Rwanda

The most commonly cited challenging topic was dose-response curve analysis, flagged by multiple participants as conceptually demanding. This suggests that future editions should allocate additional time and working examples to quantitative pharmacology.

*The session on dose-response curves was particularly challenging for me. It highlighted an area where I need to deepen my understanding.*

Kehinde O. Adeniji, Nigeria

*The session on binding curves proved the most challenging. Working through the exercises with peers solidified my understanding.*

Elias Chipofya, South Africa

## Networking, collaboration, and mentorship

A core goal of the school was to foster interdisciplinary collaboration among African researchers who rarely meet in person. The residential format – with participants, faculty, and organizers all living and working together for the duration – was designed to maximize sustained relationship building. All 13 participants described networking as a meaningful dimension of the experience.

*I especially valued connecting with Mr. Louis Gadaga regarding potential collaboration.*

Elias Chipofya, South Africa

*Group discussions and presentations provided a platform to engage with peers and foster lasting connections, opening opportunities for future collaborations.*

Rosa M. Chinheya, PhD, South Africa

Faculty accessibility outside formal sessions was consistently praised:

*The faculty was highly knowledgeable and always available for questions and discussions, both during breaks and after lectures.*

Rosa M. Chinheya, PhD, South Africa

The presence of African women scientists in faculty leadership roles was noted by several participants as particularly meaningful, providing visible role models for early-career women researchers:

*As an African woman scientist, I was inspired by the stories of Dr Rachael Dangarembizi and Dr Oritoke Okeowo. Their resilience and achievements motivated me to strengthen my own approach to research and perseverance.*

Fatma Zahra Sakhri, PhD, Algeria

*The 2nd NRDD significantly impacted my professional development, and I am already sharing the acquired knowledge through workshops and mentorship programs at my university.*

Victor Bassey Archibong, PhD, Rwanda

## Cultural and social exchange

A fourth goal of the school was to enrich the learning experience through cultural and social exchange. With participants from eight countries across North, West, East, and Southern Africa, the school was a convergence of national cultures, languages, and lived experiences. Many participants had not previously visited Southern Africa, and the program offered their first encounter with Zimbabwean culture, cuisine, and language:

*The cultural exposure was unforgettable. From visiting local shops to enjoying traditional performances, I immersed myself in Zimbabwe's rich heritage. I even learned some Shona vocabulary, adding a personal touch to the experience.*

Rhoda Mama Kolo, Nigeria

*Beyond academics, the experience offered a unique cultural exposure, from exploring local shops to enjoying Zimbabwean cuisine. The program's balance of intense study and cultural exploration made it enriching.*

Fatma Zahra Sakhri, PhD, Algeria

*The culinary experience in Zimbabwe was an exploration of local flavors, with dishes like sadza offering a taste of the region's culture.*

Kehinde O. Adeniji, Nigeria

The cross-cultural interactions that occurred during shared meals, excursions, and informal gatherings were an important complement to the formal scientific program. Participants from Francophone countries (Algeria, Morocco, Cameroon) and Anglophone countries across Eastern and Southern Africa navigated interactions in English, and several reported that the multilingual, multicultural environment strengthened their communication skills. These informal exchanges reinforced the broader mission of the school: to build a pan-African community of researchers committed to neuroscience and drug development on the continent.

## Logistical aspects and the role of setting

The residential format and choice of venue contributed meaningfully to the school's success. Manna Resorts, situated outside the Harare city center in a setting characterized by indigenous wildlife and lush greenery, provided an immersive environment that facilitated focused learning and sustained social interaction. The on-site accommodation eliminated commuting and created cohesive conditions for learning:

*The venue, Manna Resorts, was the perfect setting — calm and serene, located outside the CBD, with zebras roaming nearby. The food, accommodation, and hotel staff were all excellent.*

Rosa Chinheya, PhD, South Africa

*The venue provided an immersive experience, surrounded by lush greenery, indigenous wildlife and beautifully crafted traditional structures, providing a peaceful and inspiring atmosphere for scientific discussions.*

Wanyu Yuwong, PhD, Cameroon

*The workshop location greatly enhanced my learning experience. Set amidst breathtaking natural scenery, with wildlife and diverse flora, the environment was calming.*

Rhoda Mama Kolo, Nigeria

The school benefited from strong institutional support from IBRO and the University of Zimbabwe, enabling smooth coordination of participant travel, accommodation, and teaching materials. However, the most consistently cited area for improvement was the intensive pace of the program, with several participants noting that hands-on sessions felt compressed:

*I felt my brain was going to explode as the amount of information it was absorbing was voluminous.*

Victor Bassey Archibong, PhD, Rwanda

This feedback indicates that future editions should either extend the program duration or reduce the breadth of topics covered in favor of deeper engagement and more practical exercises.

## Reflections and future directions

The participant reflections collectively demonstrate that the 2024 IBRO-funded School on NRDD substantially achieved all four of its stated goals. Participants arrived with varied backgrounds and left with a shared foundation in neuropharmacological principles, strengthened research skills, new professional relationships, and enriched understanding of their pan-African scientific community.

Key recommendations for future editions include (1) extending the program from 1 week to 10 days to allow more time for practical exercises and consolidation of quantitative material; (2) incorporating supervised laboratory sessions alongside conceptual instruction; and (3) establishing a structured post-program follow-up mechanism – such as a participant alumni network or shared resource repository – to sustain the collaborations initiated during the program.

More broadly, the success of this school underscores the importance of discipline-specific, regionally focused research training as a complement to general university pharmacology education. The combination of African scientific context, active learning, mentorship from African faculty role models, pan-continental networking, and cultural exchange produced an experience that participants consistently described as transformative in ways that conventional coursework cannot replicate.

## Conclusion

The 2nd School on NRDD provided 13 early-career researchers from eight African countries with a rigorous and contextually relevant foundation in neuropharmacological research, with a particular emphasis on drug discovery from African natural products. Through a curriculum that integrated didactic lectures with active-learning methodologies, and a residential format that promoted sustained social interaction, the school achieved its goals of scientific capacity building, professional networking, mentorship, and cross-cultural exchange.

Participants' written reflections indicate a meaningful impact on their understanding of pharmacokinetics, experimental design, lead compound identification, and scientific communication. The school also fostered new pan-African research collaborations and provided participants with role models and mentors within the African scientific community. Areas for improvement include more hands-on practical time, additional worked examples in quantitative pharmacology, and mechanisms for sustaining collaboration after the program ends.
